# Reproductive Biology and Germination Ecology of *Phytolacca acinosa* in Its Secondary Range

**DOI:** 10.3390/plants15091362

**Published:** 2026-04-29

**Authors:** Aleksandra V. Stogova, Aleksandr A. Ivanovskii, Ekaterina V. Tkacheva, Marianna A. Zueva, Aleksandr K. Mamontov, Yulya. K. Vinogradova, Olga V. Shelepova

**Affiliations:** 1 Laboratory of Natural Flora, N.V. Tsitsin Main Botanical Garden of Russian Academy of Sciences, Botanicheskaya 4, Moscow 127276, Russia; 2 Faculty of Biology, Shenzhen MSU-BIT University, International University Park Road 1, Dayun New Town, Longgang District, Shenzhen 517182, China; 3 Faculty of Biology, Lomonosov Moscow State University, Leninskiye Gory 1, Moscow 119991, Russia; 4 Research Department of Experimental Biology and Plant Pathology, N.V. Tsitsin Main Botanical Garden of Russian Academy of Sciences, Botanicheskaya 4, Moscow 127276, Russia

**Keywords:** biological invasions, seed dormancy, endozoochory, cold stratification, cold storage, range edge, reproductive output

## Abstract

*Phytolacca acinosa* Roxb., a perennial herb native to East Asia, is increasingly naturalizing in Europe, yet its reproductive ecology in the secondary range remains poorly understood. This study evaluated seed productivity across central and edge populations in the secondary range, fruit and seed morphometrics, and germination responses to cold storage, acid scarification (simulating bird endozoochory), and light exposure. Fruit production per raceme was influenced by an interaction between insolation and range position: reduced insolation increased fruit set in central populations but decreased it at the range edge. Raceme number per shoot was lower in spontaneous plants compared to cultivated ones. Fresh seeds exhibited strong dormancy with no germination without scarification. Acid scarification significantly enhanced germination, particularly with light exposure, reaching up to 55%. Cold storage did not increase germination percentage but accelerated germination of scarified seeds under light, reducing median germination time from 24 to 21 days. Compared to the congeneric *P. americana*, *P. acinosa* shows more stringent dormancy requirements. We conclude that *P. acinosa* retains deep seed dormancy in its secondary range and relies on bird-mediated endozoochory for both dispersal and dormancy release. At the northern range edge, reduced plant vigor and lower raceme numbers are partially offset by increased flower production per raceme, though fruit set remains constrained. The species does not exhibit the simplified germination requirements often associated with successful invaders; instead, its invasion success appears driven by a bet-hedging strategy combining persistent seed banks with specific dormancy-breaking cues.

## 1. Introduction

Biological invasions represent a major driver of global environmental change, with profound ecological and economic consequences [1,2]. Among introduced plants, those with economic value—such as ornamentals, crops, or medicinal species—are particularly likely to establish self-sustaining populations outside their native ranges, as repeated introductions and widespread cultivation increase propagule pressure and facilitate escape from human care [3,4]. Plants that naturalize from cultivation often cause greater environmental and economic damage than species introduced through other vectors [5,6], emphasising the need for targeted risk assessment of widely cultivated taxa. Under ongoing climate change, shifts in temperature regimes, growing season length, and resource availability are expected to alter the dynamics of biological invasions, potentially modifying species’ reproductive success and range limits [7,8]. Understanding how invasive plants respond to these climatic gradients is therefore critical for predicting future spread.

A key determinant of invasion success is the ability to establish self-sustaining populations in novel environments, where reproductive biology and germination ecology critically influence the speed and extent of range expansion [9,10]. Seed dormancy is particularly important in this context, as it controls the timing of germination and mediates the balance between immediate establishment and long-term persistence in the soil seed bank [11]. While reduced dormancy is often considered advantageous for rapid colonization, allowing seeds to germinate as soon as conditions become favorable [12], an alternative strategy involves the maintenance of persistent seed banks combined with specific dormancy-breaking cues. Such strategies enable temporal spreading of germination (bet-hedging) and may enhance establishment success in unpredictable or heterogeneous environments [13,14,15].

The expression of germination traits in invasive species is further shaped by interactions between evolutionary history, dispersal mechanisms, and local environmental conditions [13,15]. In particular, endozoochory—the dispersal of seeds through animal digestive systems—can play a dual role by both transporting seeds and facilitating dormancy release through mechanical and chemical scarification [16,17,18,19]. The effectiveness of such mechanisms may vary between native and secondary ranges, leading to shifts in germination requirements and recruitment dynamics.

Another important dimension of invasion biology is variation in reproductive performance across the species’ range. Populations at the expanding edge of the range often experience suboptimal environmental conditions, which may reduce growth and reproductive output [20,21,22,23,24,25,26]. However, invasive species may exhibit compensatory responses, such as increased reproductive allocation or enhanced dispersal ability, which can partially offset these constraints and facilitate further spread [27,28]. Investigating how reproductive traits and germination responses vary between central and edge populations is therefore critical for understanding range expansion processes.

The genus *Phytolacca* is of particular interest to invasion biology because it includes species with valuable economic and pharmacological properties that have become invasive outside their native ranges. A well-studied example is *Phytolacca americana* from eastern North America, which has been widely introduced to Europe since colonial times [29] and is now recognized as a successful invader. This species exhibits high germination following cold stratification and broad tolerance to environmental conditions, traits that may contribute to its invasiveness [30]. More broadly, species of this genus have long been considered potential weeds due to both intentional and unintentional introductions [31,32]. A similar pattern is now observed for *P. acinosa* (Indian pokeweed), a perennial herbaceous plant native to East Asia that is increasingly recorded in the alien flora of Europe [33,34,35,36]. The species is primarily associated with urban and suburban habitats, where it spreads from cultivation and forms spontaneous populations. Despite its increasing distribution, the reproductive ecology of *P. acinosa* in its secondary range remains insufficiently studied, particularly in comparison with the closely related *P. americana*, with which it is often confused [34,37,38].

Reproduction of *P. acinosa* occurs only by seed, and scientific information on its reproductive biology in the secondary range remains insufficient. Frugivorous birds play a key role in seed dispersal [39], attracted by the shiny black-purple fruits and consuming the indigestible seeds together with the succulent pericarp. The importance of seed autotoxicity is very significant for dispersal [16] and successful naturalization, as it enables the formation of a soil seed bank that retains viability for decades [40], and germination can be paused until the most favorable conditions arise. The probability of seed dispersal before germination increases the species’ chances of colonizing a new territory, as is well demonstrated for this genus [32].

Seeds of *Phytolacca* are characterized by physical dormancy imposed by a thick seed coat, which can be alleviated by chemical scarification simulating passage through the digestive tract of frugivorous birds [16,17,18,19]. In the native Himalayan range of *P. acinosa*, freshly harvested seeds exhibit 100% viability but are deeply dormant, failing to germinate under control conditions [41]. Acid scarification (e.g., concentrated H_2_SO_4_) significantly improves germination, with optimal results achieving up to 85% germination [42,43]. Other chemical treatments, including GA_3_, KNO_3_, and thiourea, have also been shown to promote germination, though to a lesser extent [41,44]. Notably, seeds of *P. acinosa* stored under ambient conditions exhibit a peak in germination after 12 months of dry storage, followed by a gradual decline, with complete loss of viability after 30 months [41], a pattern characteristic of species with non-deep physiological dormancy [11,45]. In contrast, the only available study from the invasive range in Europe reported extremely low germination of fresh *P. acinosa* seeds across all fruit ripeness stages, with only a single seed germinating out of 450 tested [34]. This discrepancy raises important questions about the role of seed dormancy and the effectiveness of dormancy-breaking treatments for *P. acinosa* populations in the secondary range.

Understanding the reproductive strategies of invasive plants is essential for predicting their spread and for developing effective management protocols [46]. Knowledge of seed productivity, morphological variation, and dormancy-breaking requirements across the secondary range can inform eradication efforts by identifying the conditions under which seed banks are replenished and germination is triggered [34]. For *P. acinosa*, which is gradually spreading in Europe and has the potential for further range expansion [34,47], such information is critically needed but currently lacking.

The present study aims to fill this gap by evaluating (1) seed productivity of *P. acinosa* in different parts of its invasive range, (2) morphometric parameters of fruits and seeds in the secondary range, and (3) the germination capacity of fresh seeds and its dynamics depending on storage duration and conditions, including the effect of cold storage and acid scarification as a proxy for endozoochory. We tested the hypothesis that seeds of *P. acinosa* in the invasive range require dormancy-breaking treatments, particularly acid scarification, to achieve high germination percentages, reflecting the species’ adaptation to bird-mediated dispersal and its evolutionary history in the native range. By integrating data on reproductive output, seed morphology, and germination ecology across different populations, this study seeks to provide a comprehensive understanding of the factors contributing to the invasion success of *P. acinosa* in its secondary range.

## 2. Materials and Methods

### 2.1. Study Species

*Phytolacca acinosa* Roxb. (Phytolaccaceae) is a perennial herbaceous plant native to East and Southeast Asia, including regions of the Himalayas [41]. The species has been widely introduced outside its native range as an ornamental species and is increasingly recorded as naturalized in Europe, particularly in urban and suburban environments [33,34,35,36]. It is currently included in alien species lists in several European countries and is considered potentially invasive [33,34].

Within Europe, *P. acinosa* often co-occurs with the closely related *Phytolacca americana* L., a more extensively studied and generally more aggressive invasive species [34,37]. The two species are frequently misidentified due to overall morphological similarity [34,38]. However, *P. acinosa* can be distinguished by its erect, densely flowered racemes, typically bearing eight stamens and eight carpels, as well as by its ribbed fruits with clearly separated carpels that persist at maturity [35,48,49,50].

Reproduction in *P. acinosa* occurs exclusively by seeds. The species produces fleshy, dark purple fruits that are attractive to frugivorous birds, which act as the primary dispersal agents [39]. Seeds are ingested together with the pulp and subsequently dispersed over potentially long distances. Passage through the digestive tract may facilitate germination by weakening the seed coat, thereby contributing to dormancy release [16,17,18,19].

### 2.2. Study Sites Across the Range

To study the parameters of seed productivity (number of fruits per raceme, number of racemes per shoot), 10 populations of *P. acinosa* from different parts of the secondary range were studied (Table 1). Number of racemes per shoot was counted in the course of a filed survey immediately in the field (total number of studied shoots = 96). On the contrary, number of fruits per raceme was counted in the laboratory (total number of studied racemes = 57).

For each population, insolation was assessed in the field as a categorical variable based on the combined effect of canopy closure and shading from surrounding urban buildings. Sites were classified as low-, medium-, or high-insolation depending on the degree of direct sunlight exposure during the day, considering both tree canopy cover and shade cast by tall urban structures. In addition, plant status (management) was recorded as either cultivated or spontaneous.

For the purposes of analysis, populations were additionally classified according to their position within the species’ secondary range as either central or edge populations. This classification was based on the geographic distribution of the studied populations within the currently known invaded range. Populations located in the northernmost part of the studied distribution area (e.g., Yaroslavl region) were considered edge populations, whereas populations from the Moscow region were treated as central populations representing the core of the secondary range. This classification reflects the current northern expansion limit of the species at the East European Plain, with edge populations occurring under cooler climatic conditions and shorter growing seasons.

### 2.3. Mass and Water Content Measurements and Seed Morphometry

For detailed morphometric analysis, seeds of *P. acinosa* were collected from two spontaneous populations in Moscow (MOS1, September 2024; MOS2, September 2025). At the end of the fruiting period, mature fruits were sampled from the lower and middle parts of the inflorescences. Only fully developed fruits with a dark purple coloration and maximum size were selected for further analysis.

Fresh and air-dry masses of fruits and individually cleaned seeds were measured using an analytical balance (model OHAUS AR2140 (Ohaus Corporation, Pine Brook, NJ, USA), precision 0.1 mg). Water content (%) was calculated as the loss in mass relative to the initial fresh mass.

Seed size (length and width) was measured at 30× magnification, using a digital microscope Keyence VHX-1000E (Keyence Corporation, Osaka, Japan).

### 2.4. Germination Experiments

Three germination experiments were conducted (mature fruits were collected from MOS2 population, Table 1):

(1) With freshly collected fruits;
(2) With intact seeds after a storage period;
(3) With cleaned seeds after a storage period.

In the second experiment, only intact seeds were tested. In the first and third experiments, seeds were manually cleaned and then subjected to experimental treatments. Seed extraction was performed after fruit maceration using a mesh sieve (mesh size of 3 mm). The seeds were then washed under running tap water to remove pericarp residues.

In all three experiments, seeds were exposed to different insolation conditions (dark vs. light).

Experiment design for seeds from freshly collected fruits is shown in Table 2. Each Petri dish contained 25 seeds, resulting in 75 seeds per treatment (3 replicates).

For two experiments after a storage period, a portion of the dried fruits and seeds was stored in paper bags at room temperature for six months. Another portion was stored at +5 °C for six months to provide cold storage. We refer to these storage condition treatments as “cold” and “warm” storage, respectively. Dry storage was used to standardize seed condition prior to experimental treatments and to simulate potential post-dispersal desiccation in urban environments, where fruits may remain exposed before incorporation into the soil. However, we acknowledge that this may not fully reflect natural overwintering conditions.

To simulate exposure to digestive acids during endozoochory, seeds were soaked in concentrated sulfuric acid (95–98% H_2_SO_4_) for 10 min and then rinsed with distilled water three times (5 min each) [51]. This experimental factor is referred as “acid scarification” with two alternatives: “acid” and “no treatment”.

Experiment designs for both cleaned and intact seeds after a storage period are shown in Table 3. Each Petri dish contained 20–25 seeds, resulting in 64–100 seeds per treatment.

During the experiment, fungal contamination was observed in Petri dishes with intact seeds after cold storage. Therefore, an additional treatment with potassium permanganate (KMnO_4_) was introduced. Seeds were soaked in 1% solution of KMnO_4_ for 10 min without subsequent rinsing.

After the described preparations, seeds were placed in 9 cm glass Petri dishes lined with sterilized filter paper and moistened with 4 mL of distilled water with three replicates per treatment. Germination tests were conducted under two illumination conditions: light and darkness. Petri dishes assigned to the light treatment were placed under a light source (Full Spectrum LED Grow Lamp E27—SANSI 10 W (Zhejiang Leiao Import& Export Co., Ltd., Wenzhou, China)) with a photoperiod of 10 h light/14 h dark. Dishes assigned to the dark treatment were kept in complete darkness. All germination experiments were conducted at room temperature.

Seeds were monitored daily for germination. Germination was assessed based on the following stages: (i) rupture of the seed coat, (ii) radicle emergence, and (iii) cotyledon appearance. Germination was recorded at the stage of radicle emergence.

Seedlings were removed from Petri dishes upon cotyledon emergence to prevent potential autotoxic effects. Distilled water was added as needed to maintain moisture.

The germination experiment lasted for 62 days.

### 2.5. Data Analysis

Seed productivity was studied by means of two proxies: number of fruits per raceme and number of racemes per shoot. Because the racemes, collected for the laboratory investigation of fruit number, could not be linked to the specific shoots from the field survey of raceme number, the data could not be merged for multivariate analysis. Consequently, both the number of fruits per raceme and the number of racemes per shoot were analysed as two univariate variables.

To assess the effect of environmental factor (habitat insolation level) and population characteristics (plant status (management) and range position), we employed generalized liner models, where these free categorical predictors were treated as fixed effects. The number of fruits per raceme was analyzed using generalized linear models with a negative binomial error distribution and a log link function to account for the detected overdispersion in count data. The number of racemes per shoot was analyzed using a Poisson generalized linear model, as no overdispersion was detected. The full models included range position, insolation, plant status, and the interaction between range position and insolation. Model selection was based on the statistical significance of predictors and their biological interpretability. The sampling design was unbalanced, due to the low number of accessible population at the northern margin of the secondary range of *P. acinosa* and lack of spontaneous populations from habitats with a high level of insolation in our field survey. Therefore, some interaction terms could not be estimated for all factor combinations.

In germination experiments, a generalized logistic regression model was used for total germination proportion data with initially three fixed factors (acid scarification, insolation, and cold storage), since no overdispersion was detected. Model selection was based on the significance of predictors. For modelling of germination dynamics, non-linear 4–parameter logistic model was used, with parameter α set to zero (a lower horizontal asymptote).

All statistical analyses were performed in R (versions 4.3.2 and 4.4.0) [52]. Fitting of the generalized linear models with a negative binomial error distribution was made by means of the “glm.nb” function from the R package MASS [53]. The Poisson generalized linear model was fitted by means of the “glm” function of the R “stats” package [52]. Overdispersion in the data was tested with the R package “performance” [54]. Fitting of non-linear 4-parameter logistic models and subsequent visualisations were made by means of the R package “drda” [55]. Backward variable selection procedures were executed by means of the function “drop1” [52]. Tests of term significance was executed by means of the “Anova” function from the R package “car” [56]. Predicted marginal means and their 95% confidence intervals were calculated using the “ggeffects” package [57]. All visualisations, excepting for non-linear 4-parameter logistic models, were performed by means of the “ggplot2” R package [58].

## 3. Results

**Figure 1:**
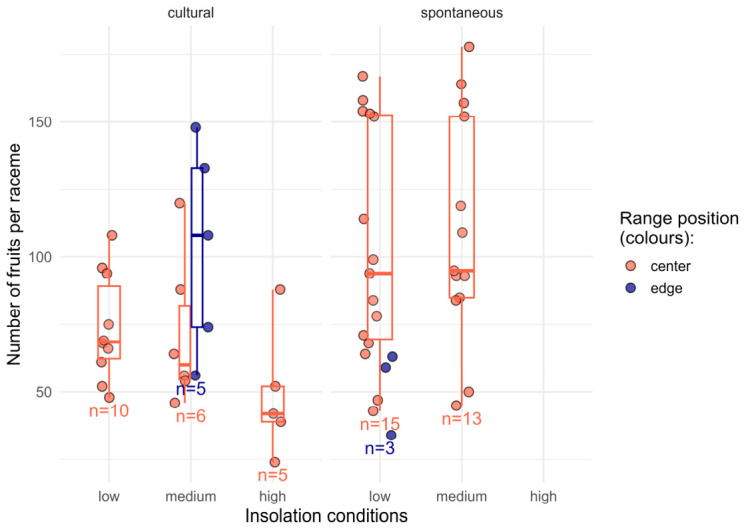
Variation in the number of fruits per raceme across different groups of studied populations (sample sizes of groups are shown below the respective groups). The circles show the raw values. The left and the right panes refer to cultural and spontaneous populations respectively (plant status). Five-number statistics (boxplots) are shown for the samples with *n* > 3. Outliers were not calculated due to small sample size in each group. Boxplot widths are adjusted for visualisation purposes only.

The number of fruits per racemes across all studied populations (Figure 1) ranged from 24 to 178 (Q1 = 56, the median = 84, Q3 = 109; *n* = 57). The lack of spontaneous populations from habitats with a high insolation level in our field survey led to an unbalanced design that challenged testing of the regression models.

The number of fruits per raceme was significantly influenced by insolation, plant status, and its interaction with range position. In the regression model, which included both insolation and plant status, plants growing under low and medium insolation in central populations produced significantly more fruits per raceme compared to those under high insolation (*p* < 0.05 in the regression model). Plant status also had a significant effect on the number of fruits per raceme (the explanatory term significance *p* < 0.05 in the same regression model), with spontaneous individuals producing on average 45% more fruits per raceme than cultivated ones (*p* < 0.05 in Kruskal–Wallis test, *n* = 26 and 31, respectively). This non-parametric test was used due to the non-normal distribution of the spontaneous population sample (Shapiro–Wilk test).

Small sample size for edge populations limited the statistical testing of factor interactions in the models, which included the range position. In the raw data, the effect of light conditions differed between central and edge populations. In the edge populations, higher fruit production was registered under higher insolation. In the central populations, this effect was absent. The interaction term for medium insolation at the range edge could not be estimated due to the absence of observations for this combination.

**Figure 2:**
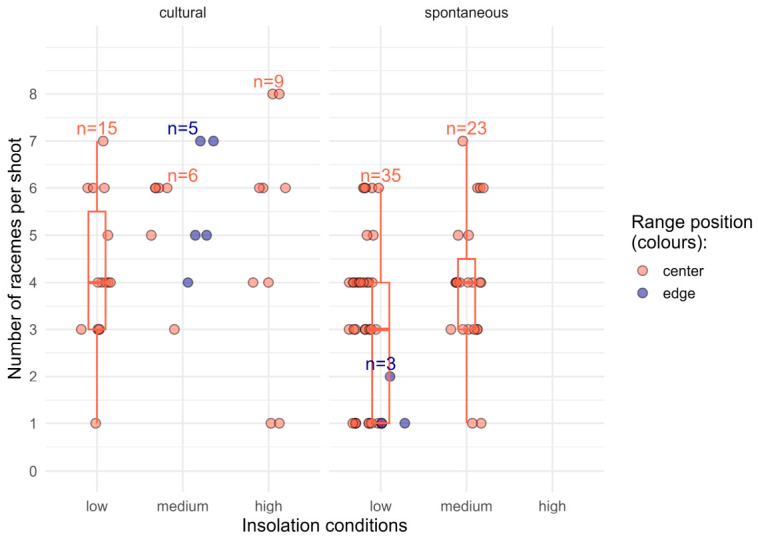
Variation in the number of racemes per shoot across different groups of studied populations (sample sizes of groups are shown above the respective groups). The circles show raw values. The left and the right panes refer to cultural and spontaneous populations respectively (plant status). Five-number statistics (boxplots) are shown for the samples with *n* > 10. Outliers were not calculated due to the small absolute range within each group.

The number of racemes per shoot across all studied populations (Figure 2) ranged from 1 to 8 (Q1 = 3; the median = 4; Q3 = 5.3; *n* = 96).

The number of racemes per shoot was significantly affected only by plant status (*p* < 0.01 in the regression model with three factors). Spontaneous plants produced approximately 24% fewer racemes per shoot compared to cultivated individuals (*p* < 0.001 in Kruskal–Wallis test, *n* = 35 and 61, respectively). This non-parametric test was used due to the non-normal distribution of the spontaneous population sample (Shapiro–Wilk test). Neither insolation nor range position had a significant effect on raceme number (*p* > 0.05 in the same regression model).

Morphometric characteristics of fruits and seeds are summarized in Table 4.

Fully developed fruits of *P. acinosa* consistently contained eight carpels, corresponding to the typical species morphology. Inflorescences (racemes) comprised up to 180 flowers; however, flowers located in the upper part of the raceme frequently failed to develop into fruits, contributing to the observed proportion of incompletely developed carpels.

No germination was observed in freshly collected seeds, regardless of light conditions (light or darkness). Removal of the drupelet pericarp (seed cleaning) from freshly collected fruits also did not result in germination. These results indicate the presence of seed dormancy in *P. acinosa*.

All treatments of fresh seeds and intact seeds after storage showed extremely low germination rate: none of the 300 fresh seeds germinated and only a single seed germinated across all replicates with intact seeds for either cold or warm storage treatments (350 seeds). Due to this negligibly low germination rate, further statistical analysis of this experiment was not performed.

Before the germination experiment, the cleaned seeds were stored under either cold or warm conditions, which was the first factor assessed in the germination experiment. The other two experimental factors were acid scarification before the experiment and illumination conditions (dark vs. light) during the germination. The former mimicked passage through a digestive system of frugivorous animals. The latter simulated different levels of insolation at natural germination sites. These three factors formed a fully replicated factorial design of the germination experiment with 2 × 2 × 2 = 8 experimental treatment combinations (3 replicates per combination).

**Figure 3:**
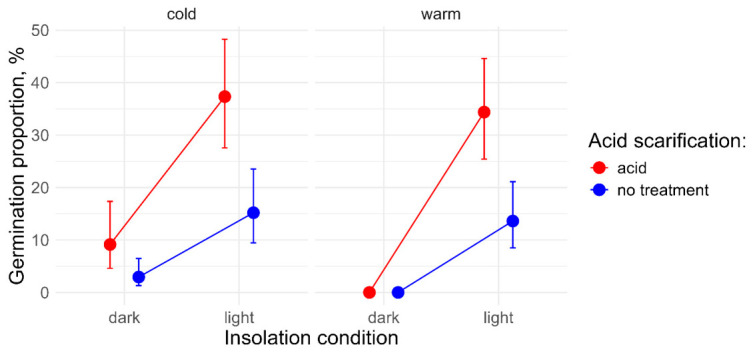
The effects of light conditions, cold storage, and acid treatment on the final germination proportion of cleaned seeds of *P. acinosa*, obtained from the logistic regression model. The points represent the predicted germination proportions, and the error bars indicate the respective 95% confidence intervals. The lines show the effect of insolation conditions. The effect of acid scarification is visualised by means of colours. The panel names “cold” and “warm” refer to the respective storage condition treatments. No confidence intervals could be calculated for two combinations of experimental treatments, where zero variation in total germination was found.

In different treatment combinations, total germination differed from 0 (n = 8) to 55% (n = 1) (Supplementary Datafile S3). The variable selection procedure returned all three primary factors (*p* < 0.001 in each case) and an interaction between cold stratification and insolation (*p* < 0.01) as significant explanatory variables in the logistic regression model (Figure 3). The highest germination probability occurred under “light” illumination treatment following “acid” scarification treatment. For two combinations of experimental treatments (“dark” insolation conditions and “warm” storage period), the germination rate was zero irrespective of acid scarification treatments (n = 3 for both). This made it impossible to draw a conclusion about the statistical significance of the respective marginal effects.

Since the “dark–warm” treatment combination prevented the estimation of marginal effects, we subsequently assessed the influence of cold storage and light exposure separately. This was done by excluding from the original three-way model an entire factor where zero germination was observed in any specific treatment combination.

**Figure 4:**
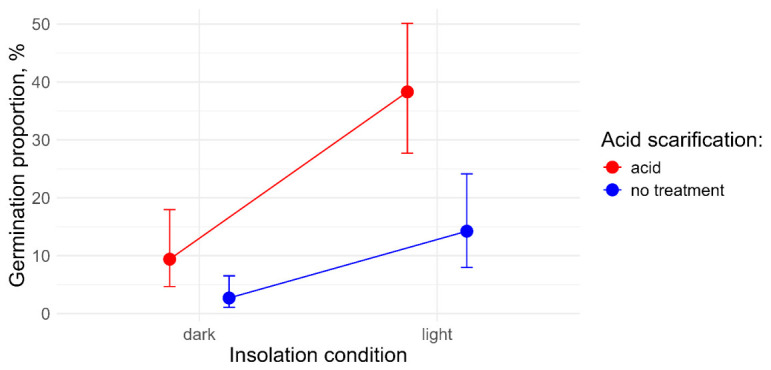
The effects of acid scarification and insolation on total germination after uniform cold storage.

By removing the storage factor from the analysis, we obtained a two-way model to assess the effects of illumination and acid scarification after uniform cold storage (Figure 4).

The final model included both primary factors; the interaction term was excluded by the variable selection procedure. Both the acid scarification and the illumination showed significant positive effects on total germination (*p* < 0.001), most prominent for the “light” illumination condition after acid scarification.

**Figure 5:**
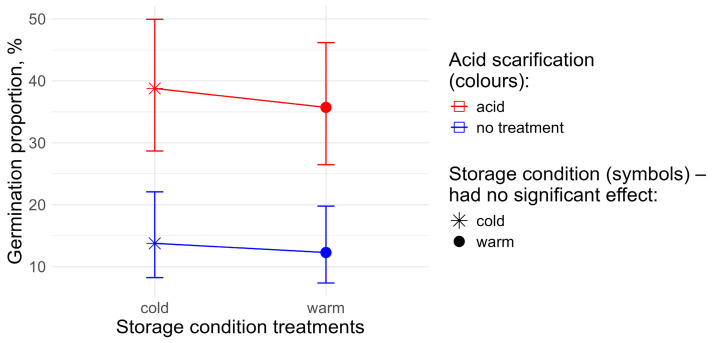
The effects of cold storage and acid scarification on total germination under uniform “light” illumination condition.

By excluding the illumination factor from the analysis, we obtained another two-way model to evaluate the effects of cold storage and acid scarification under a uniform “light” illumination condition (Figure 5).

The cold storage had no significant effect; therefore, the final model was a one-way model with the acid scarification as the sole explanatory factor (*p* < 0.001), with an odds ratio of 3.97 for the acid treatment. Consequently, cold storage did not increase germination under light exposition after acid scarification.

**Figure 6:**
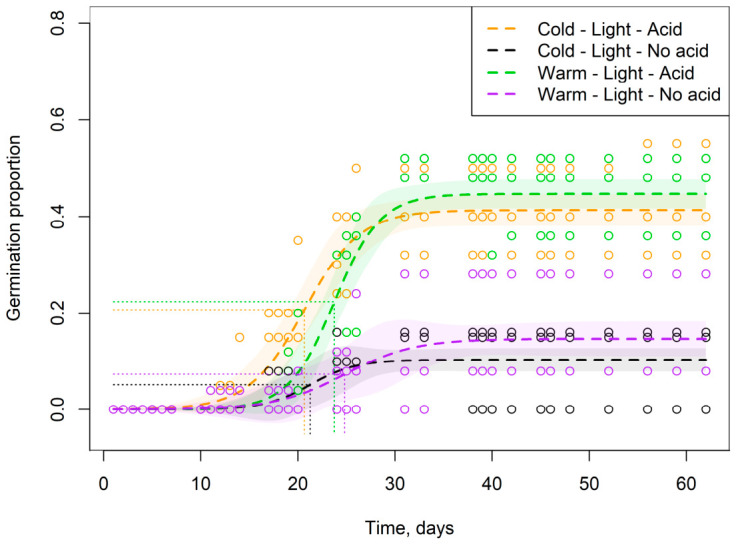
Germination dynamics of *P. acinosa* seeds under different treatment combinations with non-zero germination. The open circles mark the individual data points. The curves represent cumulative germination over time. Shaded areas indicate 95% confidence intervals. The dotted lines show the points at which half of the total germinated seeds had emerged (median germination time, T_50_).

As mentioned above, two of the eight combinations of experimental treatments yielded zero germination in every replicate. Additionally, two other combinations showed extremely low germination rates with only 1 or 2 seeds germinated in each replicate within the experimental unit. The remaining four combinations displayed higher germination rates and were used to analyse the germination dynamics (Figure 6).

**Figure 7:**
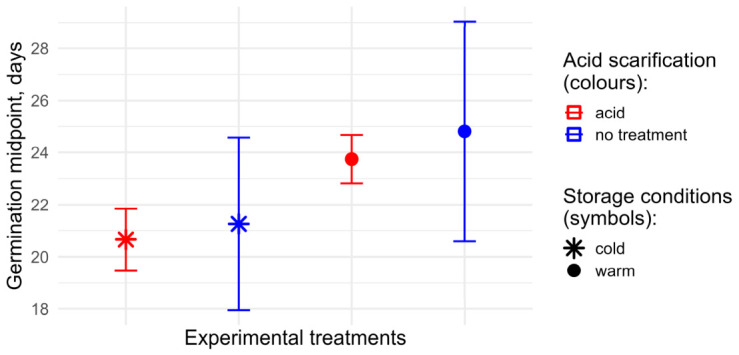
The median germination time estimates from the fitted germination curves. The points represent the parameter φ of the fitted logistic curves (T_50_ points). The whiskers show 95% confidence intervals of the parameter values.

Aside from the enhancing effect of the acid scarification on germination under light exposure (the two top curves in Figure 6) mentioned above, we detected a subtle but significant difference in the median germination times between two “acid” treatments (Figure 7). Specifically, for acid-scarified seeds, the 95% confidence intervals of the estimated median germination times did not overlap, which we interpret as evidence of an enhancing effect of cold storage (ca. 21 vs. ca 24 days).

## 4. Discussion

The present study demonstrates that different components of reproductive output in *Phytolacca acinosa* are regulated by distinct environmental and biological factors, reflecting a complex and flexible reproductive strategy across the species’ secondary range.

### 4.1. Reproductive Output and Its Environmental Drivers

The number of racemes per shoot was primarily determined by plant status, with spontaneous individuals producing significantly fewer racemes than cultivated plants. This pattern likely reflects differences in resource availability and competitive environment. Cultivated plants typically experience more favorable conditions, including reduced competition and enhanced water and nutrient supply, whereas spontaneous individuals may allocate more resources to maintenance and stress tolerance rather than to structural components of reproduction [13,15].

In contrast, fruit production per raceme was strongly influenced by insolation and its interaction with range position. In central populations, reduced insolation was associated with increased fruit production, whereas at the range edge the same conditions led to decreased reproductive output. This opposing response suggests that the effect of light availability is highly context-dependent and mediated by local environmental constraints. In central populations, moderate shading may alleviate abiotic stress, such as excessive radiation or water loss, thereby enhancing reproductive performance. At the northern range margin, however, reduced light availability likely further limits carbon assimilation under already suboptimal climatic conditions. Given that *P. acinosa* is native to warmer regions of South and Southeast Asia [41], such conditions may constrain growth and reproductive processes, particularly at the expanding edge of the secondary range.

This pattern aligns with the well-documented tendency for many species to exhibit reduced reproductive performance toward the periphery of their range, often attributed to suboptimal environmental conditions and demographic constraints [20,21,22,23,24,25,26]. However, invasive species may occasionally deviate from this pattern, and in some cases edge populations experience selection for enhanced dispersal ability or reproductive output [27,28]. In *P. acinosa*, the absence of a positive effect of high insolation on fruit production in central populations, contrasted with its strong positive effect at the range edge, suggests that light availability becomes a limiting factor only under more stressful climatic conditions.

### 4.2. Seed Production and Reproductive Investment

The high overall fruit set (91%) indicates that pollination is generally not a limiting factor in the studied populations. However, the relatively low proportion of fully developed carpels (68%) suggests that resource limitation or selective abortion may constrain seed maturation. Such patterns are common in plants with high reproductive output, where not all ovules develop into viable seeds [11].

The observed seed mass (102 mg per 100 seeds) and size are comparable to values reported from the native range [41], indicating that seed provisioning is not substantially altered in the invasive range. This suggests that differences in invasion success are unlikely to be driven by changes in seed size or maternal investment, but rather by post-dispersal processes such as dormancy and germination.

### 4.3. Seed Dormancy and Germination Ecology

One of the key findings of this study is the persistence of strong seed dormancy in *P. acinosa* within its secondary range. The complete absence of germination in freshly collected seeds, regardless of light conditions or pericarp removal, indicates that dormancy is intrinsic to the seed rather than imposed solely by fruit tissues. This result is consistent with observations from the native Himalayan range, where freshly harvested seeds exhibit high viability but fail to germinate without dormancy-breaking treatments [41,59].

Similarly, extremely low germination of untreated seeds after storage corroborates previous findings from the invasive range in Europe [34], suggesting that dormancy characteristics are conserved across ranges. This contrasts with the commonly proposed pattern for invasive species, where reduced dormancy facilitates rapid establishment [12]. Instead, *P. acinosa* appears to retain a conservative germination strategy characterized by stringent dormancy requirements.

Acid scarification was the most effective treatment for breaking dormancy, particularly in combination with light exposure. This finding is consistent with studies from the native range demonstrating strong positive effects of chemical scarification on germination [42,43]. The effectiveness of this treatment supports the hypothesis that passage through the digestive tract of frugivorous birds plays a key role in dormancy release [16,17,18,19], indicating that endozoochory serves a dual function by facilitating both dispersal and germination.

In contrast, cold storage alone did not significantly increase germination percentage, which is in agreement with previous studies on *P. acinosa* in the invasive range [34]. However, it reduced the median germination time of scarified seeds, suggesting that cold exposure may act as a secondary modifier of germination dynamics rather than a primary dormancy-breaking mechanism.

The requirement for light as a germination cue further distinguishes *P. acinosa* from the closely related *Phytolacca americana*. While *P. americana* exhibits high germination after cold stratification under both light and dark conditions [30], *P. acinosa* requires light for successful germination, indicating a more restricted set of suitable microsites for recruitment.

### 4.4. Ecological and Evolutionary Implications

Taken together, these results suggest that *P. acinosa* does not follow the commonly assumed invasion strategy based on rapid germination and reduced dormancy. Instead, it relies on a combination of persistent seed dormancy and specific dormancy-breaking cues, employing a dual reproductive strategy in its secondary range. On the one hand, structural and phenological adjustments—such as the context-dependent response of fruit production to insolation—may optimize reproductive success under varying environmental conditions. On the other hand, the combination of seed dormancy with mechanisms that enhance germination after dispersal (acid scarification and light exposure) promotes persistence and spread across heterogeneous environments.

Seed dormancy is widely recognized as an important adaptive trait in invasive species, as it enables temporal spreading of germination and reduces the risk of recruitment failure under unfavorable conditions [14,60]. By delaying germination, dormant seeds can persist in the soil seed bank and germinate when conditions become suitable, thereby enhancing population persistence and colonization ability [14,61]. For *Phytolacca* species, the ability to form a long-persistent seed bank has been documented previously [32,40] and is likely a key factor contributing to their invasion success.

The requirement for specific dormancy-breaking cues, such as acid scarification and light exposure, supports a bet-hedging strategy, in which germination is distributed over time and restricted to favorable conditions, reducing the risk of recruitment failure in unpredictable environments [14,60,61,62,63]. Such strategies may be particularly advantageous during range expansion, where environmental conditions are more variable and often suboptimal [13,15].

The contrasting germination strategies of *P. acinosa* and *P. americana*—the former requiring more specific cues (acid scarification and light) and the latter responding more readily to cold stratification alone—may help explain differences in their invasive dynamics. *P. americana* is generally considered more aggressive and widespread in Europe [34,37], and its more flexible germination system may contribute to its broader ecological amplitude. In contrast, *P. acinosa* may be more dependent on bird-mediated dispersal not only for seed transport but also for dormancy release, potentially limiting its spread to habitats with active frugivore populations.

### 4.5. Limitations and Future Directions

It should be noted that the field sampling design was partially unbalanced, and some combinations of factors (e.g., medium insolation at the range edge) were not represented, which limited the full evaluation of interaction effects. Nevertheless, the observed patterns are consistent and biologically meaningful, highlighting the importance of considering both environmental gradients and life-history traits when assessing plant reproductive performance in the context of biological invasions.

By integrating data on reproductive output, seed morphology, and germination ecology across different populations, this study provides a comprehensive understanding of the factors contributing to the invasion success of *P. acinosa* in its secondary range. The findings underscore that successful invasion is not driven by a single trait but rather by a combination of flexible reproductive responses and dormancy mechanisms that balance immediate establishment with long-term persistence. Comparisons with *P. americana* [30,34] highlight that even congeneric species can exhibit markedly different germination strategies, emphasizing the need for species-specific investigations in invasion biology.

In addition, the use of dry storage prior to germination experiments may not fully reflect natural overwintering conditions, where seeds are likely to remain under moist conditions.

## 5. Conclusions

At the northern edge of its range, *P. acinosa* exhibits reduced reproductive effort, producing fewer inflorescences per shoot than central populations. Although the number of flowers per inflorescence is generally higher at the range edge, the fruit set remains limited, suggesting that reproductive compensation does not fully offset the constraints imposed by shorter growing seasons and lower temperatures—factors likely to influence northward expansion under climate change.

Fresh seeds exhibit strong dormancy that persists in the secondary range, unlike patterns observed in some invasive species. This persistence of dormancy may represent a bet-hedging strategy against climate variability, allowing seed banks to persist during unfavorable seasons.

Acid scarification, which reflects adaptation to endozoochory by birds, is the primary factor enhancing germination, especially when combined with light exposure, confirming the critical role of frugivorous birds in both dispersal and dormancy release. Cold storage does not increase germination percentage but significantly accelerates germination of scarified seeds under light—an advantage at the northern range edge, where growing seasons are shorter.

*P. acinosa* exhibits stringent germination requirements in comparison with congeneric *P. americana*, relying on specific cues rather than responding readily to cold storage alone. Such differences may predict differential responses to future climate scenarios.

The studied populations do not exhibit reduced dormancy or increased germination flexibility—traits often associated with invasive species—suggesting that invasion success is driven by a risk-hedging strategy rather than by a simplification of germination requirements.

Finally, zero or very low germination across several treatment combinations highlights the need for larger sample sizes in future studies of dormant seeds, particularly when assessing responses to climate change, which require high statistical power.

## Figures and Tables

**Table 1:** Location and characteristics of the studied populations of *P. acinosa*. The primary data are shown in the Supplementary Datafiles S1 and S2.

Population Code	Location	Range Position	Management	Insolation	Geolocation	Number of Sampled Shoots	Number of Sampled Racemes
MOS1	Moscow, Khalturinskaya Street	center	spontaneous	low	55°47′58″ N 37°43′38″ E	22	11
MOS2	Moscow, Botanical Garden of the First Moscow State Medical University named after I.M. Sechenov	center	spontaneous	medium	55°44′52″ N 37°31′45″ E	23	13
MOS3	Moscow, Nansen Street	center	spontaneous	low	55°51′5″ N 37°39′11″ E	13	4
MOS4	Moscow, Bolshaya Lubyanka Street	center	culture	medium	55°45′56″ N 37°37′49″ E	6	6
MOS5	Moscow, 2nd Pavlovsky Lane	center	culture	low	55°43′4″ N 37°37′55″ E	9	4
MOS6	Moscow, Altufyevskoye Shosse	center	culture	high	55°54′28″ N 37°35′13″ E	4	—
MOS7	Moscow, 3rd Kolobovsky Lane	center	culture	high	55°46′9″ N 37°37′4″ E	4	5
OBN	Obninsk, Kaluga region,	center	culture	low	55°5′19″ N 36°36′5″ E	6	6
RYB	Rybinsk, Yaroslavl region	edge	spontaneous	low	58°9′46″ N 38°59′38″ E	3	3
BOR	Borok village, Yaroslavl region	edge	culture	medium	58°3′56″ N 38°13′58″ E	5	5

**Table 2:** Treatments of germination experiment of *P. acinosa* fresh seeds.

Insolation Treatments	Cleaned Seeds	Intact Seeds
	*n*, Petri Dishes (*n*, Seeds, in All Dishes)	*n*, Petri Dishes (*n*, Seeds, in All Dishes)
light	3 (75)	3 (75)
dark	3 (75)	3 (75)

**Table 3:** Treatments of germination experiments with *P. acinosa* seeds after storage period. The asterisks (*) show the treatment combinations in the cleaned seeds experiment, where zero germination in all three replicates (Petri dishes) was found.

Insolation Treatments	Acid Scarification Treatments	Cleaned Seeds		Intact Seeds	
		Cold Storage, *n*, Petri Dishes (*n*, Seeds, in All Dishes)	Warm Storage, *n*, Petri Dishes (*n*, Seeds, in All Dishes)	Cold Storage, *n*, Petri Dishes (*n*, Seeds, in All Dishes)	Warm Storage, *n*, Petri Dishes (*n*, Seeds, in All Dishes)
light	acid	3 (65)	3 (75)	-	-
	no treatment	3 (64)	3 (75)	-	-
dark	acid	3 (65)	3 (75) *	3 (75)	4 (100)
	no treatment	3 (70)	3 (75) *	3 (75)	4 (100)

**Table 4:** Fruit and seed traits.

Trait	Value (Mean ± SE)	Sample Size
Fruit set (%)	91 ± 1.6	15 racemes (2137 flowers)
Fully developed carpels (%)	68 ± 3.3	80 fruits (640 carpels)
Dry mass of 100 carpels (mg)	183.2 ± 2.5	60 × 5 carpels *
Water content (%)	60.7 ± 4.5	60 × 5 carpels *
Mass of 100 seeds (mg)	102.0 ± 1.1	100 × 5 seeds *
Seed length (mm)	3.50 ± 0.03	80 seeds
Seed width (mm)	2.91 ± 0.02	80 seeds

*—seeds were weighed in five replicates.

## Data Availability

The original contributions presented in this study are included in the Supplementary Materials. Further inquiries can be directed to the corresponding author.

## Supplementary Materials

The following supporting information can be downloaded at https://www.mdpi.com/article/10.3390/plants15091362/s1, Datafile S1: seed productivity data file (number of fruits per raceme); Datafile S2: seed productivity data file (number of racemes per shoot); Datafile S3: total seed germination data file; Datafile S4: seed germination dynamics data file.
